# Puberté précoce: expérience de l´unité d´endocrinologie pédiatrique à l´Hôpital d´Enfant de Rabat

**DOI:** 10.11604/pamj.2022.42.149.29289

**Published:** 2022-06-23

**Authors:** Kaoutar Rifai, Youssra El Khayat, Ahmed Gaouzi

**Affiliations:** 1 Unité d´Endocrinologie, Service de Pédiatrie II, Hôpital d´Enfants, Faculté de Médecine et de Pharmacie, Université Mohammed V Souissi, Rabat, Maroc,; 2 Service d’Endocrinologie et Maladies Métaboliques, CHU Ibn Sina de Rabat, Rabat, Maroc

**Keywords:** Puberté précoce, puberté précoce centrale, pseudopuberté précoce, Precocious puberty, central precocious puberty, pseudo-precocious puberty

## Abstract

**Introduction:**

la puberté précoce est définie par le développement des caractères sexuels avant l´âge de 8 ans chez la fille et de 9 ans chez le garçon. L´objectif de notre étude était de décrire le profil clinique, paraclinique et étiologique de la puberté précoce.

**Méthodes:**

il s´agit d´une étude descriptive rétrospective s´étalant de 1999 à 2017 à l´unité d´endocrinologie pédiatrique à l´Hôpital d´Enfant de Rabat.

**Résultats:**

quatre-vingt-dix-neuf (99) enfants ont été inclus. L´âge moyen des filles était de 4,25 ± 2,6 ans, celui des garçons était de 3,6 ans ± 1,8. Nous avons noté une prédominance féminine dans 90% (90 filles). Les signes révélateurs chez la fille étaient dominés par le développement mammaire (77,77%). Chez le garçon, le motif de consultation le plus fréquent était la pilosité pubienne (70%). Sur le plan biologique, dans les pubertés précoces centrales, le taux moyen du pic de LH après stimulation par GnRH était élevé à 17 UI/L avec un rapport moyen pic LH/ pic FSH de 1,30. Les étiologies étaient comme suit: pubertés précoces dissociées (60,60%), pubertés précoces pathologiques (39,40%). Dans cette dernière, nous avons noté une prédominance des pseudo-pubertés précoces (58,98%). Pour la puberté précoce centrale, la forme idiopathique était l´étiologie la plus fréquente chez la fille (62,5%). Alors que chez tous les garçons, une lésion du système nerveux central était objectivée.

**Conclusion:**

notre étude confirme que la puberté précoce pathologique centrale est souvent liée à une lésion du système nerveux central chez le garçon justifiant ainsi la réalisation systématique d´une imagerie cérébrale.

## Abstract (en)

**Introduction:**

precocious puberty is defined as the development of sexual characteristics before 8 years of age in girls and 9 years of age in boys. The purpose of our study was to describe the clinical, paraclinical and etiological profile of precocious puberty.

**Methods:**

we conducted a retrospective descriptive study from 1999 to 2017 in the Pediatric Endocrinology Unit at the Rabat Children’s Hospital.

**Results:**

ninety-nine children were included in the study. The average age of girls was 4.25 ± 2.6 years, while that of boys was 3.6 ± 1.8 years. There was a female predominance (90%; 90 girls). The telltale signs in girls were dominated by breast development (77.77%). In boys, the most common reason for consultation was pubic hair (70%). Biologically, in central precocious puberty, the mean peak LH level after GnRH stimulation was high (17 IU/L) with a mean peak LH to peak FSH ratio of 1.30. The causes included: dissociated early puberty (60.60%), pathological early puberty (39.40%). In the latter, we noted a predominance of precocious pseudopuberty (58.98%). Concerning the central precocious puberty, idiopathic central precocious puberty was the most common etiology in girls (62.5%). In all boys, central nervous system lesion was found.

**Conclusion:**

our study confirms that central pathological precocious puberty is often related to a lesion of the central nervous system in boys, thus justifying systematic brain imaging.

## Introduction

La puberté précoce est définie par le développement des caractères sexuels avant l´âge de 8 ans ou la survenue de ménarche avant l´âge de 9 ans chez la fille. Chez le garçon, l´apparition des caractères sexuels secondaires avant l´âge de 9 ans définit la puberté précoce [1]. Ce développement peut correspondre à une variante de la puberté normale ou à une puberté précoce pathologique [2]. Cette dernière peut être d´origine centrale hypothalamo-hypophysaire (puberté précoce vraie ou puberté GNRH dépendante) ou d´origine périphérique (pseudo puberté précoce ou puberté GNRH indépendante): testiculaire ou ovarienne ou surrénalienne [3]. La puberté précoce est le plus souvent d´origine centrale. Chez la fille, la puberté précoce centrale est idiopathique dans plus de 80% des cas. Chez le garçon, elle est due à une lésion du système nerveux central (SNC) dans 70% des cas [2]. La puberté précoce est un sujet peu étudié en Afrique, cela justifie notre revue rétrospective, portant sur 99 enfants suivis à l´unité d´endocrinologie pédiatrique à l´Hôpital d´Enfant de Rabat, et dont l´objectif était de décrire les aspects épidémiologiques, cliniques, para cliniques, et étiologiques des pubertés précoces.

## Méthodes

**Contexte de l´étude:** il s´agit d´une étude descriptive rétrospective transversale. Nous avons recruté les enfants pris en charge pour une puberté précoce à l´unité d´endocrinologie pédiatrique à l´Hôpital d´Enfant de Rabat entre décembre 1999 et décembre 2017. La collecte des données s´est faite de manière rétrospective entre janvier 2018 et mars 2018.

**Cadre de l´étude:** elle a été menée à l´unité d´endocrinologie pédiatrique à l´Hôpital d´Enfant de Rabat sur une période de 18 ans (1999-2017). Ce dernier dessert la région Rabat Salé Kénitra et tout le nord du Maroc.

**Participants:** les critères d´inclusions étaient les enfants qui avaient consulté pour une puberté ayant débuté avant l´âge de 8 ans pour les filles et avant 9 ans pour les garçons durant la période allant du 1^er^ décembre 1999 au 31 décembre 2017. Les enfants ayant une puberté avancée ont été exclus de l´étude ainsi que ceux dont les dossiers médicaux étaient sans renseignements suffisants malgré les tentatives de les compléter.

**Variables:** les paramètres recueillis ont inclus l´âge, le sexe, les antécédents de traumatisme crânien, de radiothérapie cérébrale, de méningite, de prise de stéroïdes ou de contamination par les pesticides, et la présence de cas similaires dans la famille. Les renseignements diagnostiques ont été: les stades du développement pubertaire selon la classification de Tanner et Marshall, la croissance staturale en déviations standards, et les examens complémentaires (dosages hormonaux réalisés par méthode immunométrique: testostérone, œstradiol, FSH, LH test à la LHRH, la radiographie du poignet gauche, l´échographie pelvienne, et l´IRM cérébrale).

**Source de données:** le recueil a été fait à partir des dossiers médicaux complets et exploitables.

**Collecte et analyse des données:** la collecte et l´analyse des données ont été faites à partir d´une fiche d´enquête.

**Biais:** pour réduire le risque des biais d´information et d´enregistrement, les données ont été recueillies par deux personnes et les données finales ont résulté de la fusion des listes finales.

**Taille de la population d´étude:** la taille de l´échantillon a été déterminée par le nombre des patients ayant satisfait aux critères d´inclusion sans avoir d´élément des critères d´exclusion.

**Analyses statistiques:** toutes les données ont été recueillies et l´analyse statistique s´est faite via le logiciel SPSS Smartviewer 18.0. Les variables quantitatives sont exprimées en moyenne ± écart type (distribution gaussienne) et les variables qualitatives en pourcentage et en effectif.

## Résultats

**Participants:** nous avons colligé un total de 111 patients dont 12 étaient exclus pour des dossiers incomplets. Au total, 99 enfants ont été inclus dans notre étude.

**Données descriptives:** sur une période de 18 ans, nous avons colligé 99 dossiers de patients qui répondaient à nos critères de sélection, soit une fréquence annuelle moyenne de 5,5 cas par an. Chez les filles, l´âge moyen était de 4,25 ± 2,6 ans, celui des garçons était de 3,6 ans ± 1,8. Nous avons noté une nette prédominance féminine dans 90% des cas (90 filles, 9 garçons). Pour les antécédents, 1 garçon avec une puberté précoce centrale avait un antécédent de traumatisme crânien, une fille avec prémature pubarche avait un antécédent de méningite. Une prise médicamenteuse pendant la grossesse (corticoïdes, antidépresseurs et anxiolytique) a été retrouvé dans 2 cas. Un cas similaire a été noté chez une fille présentant un bloc enzymatique partiel en 11 β hydroxylase.

### Principaux résultats

**Les données cliniques:** chez les filles, les signes révélateurs de la puberté précoce étaient dominés par le développement mammaire dans 77,77% (70 filles) suivi par la pilosité pubienne dans 31,11% (28 filles), la ménarche prématurée a été notée dans 1,11% (une seule fille). Chez les garçons, le motif de consultation le plus fréquent était la pilosité pubienne (70%) suivie de l´augmentation de la taille de la verge et du volume testiculaire (20%). L´avance staturale moyenne était de + 2,7 ± 0,8 DS.

**Les examens complémentaires:** dans les pubertés précoces centrales des filles, l´échographie pelvienne a objectivé une longueur utérine moyenne augmentée à 39 ± 4 mm. Sur le plan biologique, le taux moyen de base de LH était augmenté à 2,57 ± 1,03 UI/L. Lors du test de stimulation LH-RH, le taux moyen du pic de LH était élevé à 17 ± 5 UI/L avec un rapport moyen pic LH / pic FSH de 1,30. Chez les garçons avec puberté précoce, il y avait une accélération de la maturation osseuse avec un âge osseux moyen de 10 ± 5 ans. Sur le plan biologique, le taux moyen de testostérone était augmenté à 3,38 ± 0,9 ng/ml.

**Les profils étiologiques:** dans notre série, les étiologies étaient représentées comme suit: puberté précoce dissociée dans 59,40% (60 cas), et la puberté précoce pathologique dans 38,60% (39 cas). Dans la puberté précoce dissociée, la forme la plus fréquente était la prémature thélarche (80%) suivis de la prémature pubarche (18%) puis prémature ménarche (2%). Concernant la puberté précoce pathologique, 41,02% avaient une puberté précoce centrale (16 cas) et 58,98% avaient une pseudo puberté précoce (23 cas). Pour cette dernière, l´hyperplasie congénitale des surrénales (HCS) par bloc en 21 hydroxylase dans sa forme non classique était l´étiologie la plus fréquente dans 26,08 % (6 cas), suivie de l´HCS par bloc en 11β hydroxylase dans 21,73% (5 cas), du corticosurrénalome dans 17,39% (4 cas), du syndrome de McCune Albright dans 8,69%(2 cas), du kyste ovarien dans 8,69% (2 cas), de tumeurs de la Granulosa dans 8,69%(2 cas), de testotoxicose dans 4,34% (2 cas), et enfin du syndrome de Van Wyk Grumbach dans 4,34% (1 cas) (Tableau 1). Pour la puberté précoce centrale (16 cas), 62,5 % des filles (10 cas) avaient une forme idiopathique avec une imagerie cérébrale normale, 12,5% des filles (2 cas) avaient un hamartome hypothalamique, 6,25% (1 cas) des filles avaient un microadénome hypophysaire à LH, et 6,25% (1 cas) des filles avaient une hydrocéphalie. Pour les garçons, 12,5% (2 cas) avaient un hamartome hypothalamique (Tableau 2).

**Tableau 1:** étiologies des pubertés précoces centrales chez les 16 patients

Etiologies &	Filles	Garçons
Idiopathique	10 (62,5)	0
Hamartome hypothalamique	2 (12,5)	2 (12,5)
Hydrocéphalie	1 (6,25)	0
Microadénome à LH	1 (6,25)	0

& : exprimé en effectif et pourcentage

**Tableau 2:** étiologies des pseudo-pubertés précoces chez les 23 patients

Etiologies &	Filles	Garçons
HCS par bloc en 21 hydroxylase	2 (8,69)	4 (17,39)
HCS par bloc en 11 b hydroxylase	3 (12,50)	2 (8,69)
Corticosurrénalome	4 (17,39)	0
Syndrome de McCune - Albright	2 (8,69)	0
Kyste ovarien	2 (8,69)	0
Tumeur ovarienne (tumeur de Granulosa)	2 (8,69)	0
Testotoxicose	0	1 (4,34)
Syndrome de Van Wyk-Grumbach	1 (4,34)	0

& : exprimé en effectif et pourcentage

## Discussion

Notre étude a trouvé une nette prédominance féminine avec comme étiologie principale la puberté précoce dissociée. Pour les pubertés précoces pathologiques, notre travail avait comme particularité la dominance des pseudos pubertés précoces. Pour les pubertés précoces centrales, la forme idiopathique était la forme la plus fréquente chez la fille. Par contre chez les garçons, une lésion du système nerveux central a été objectivée dans tous les cas. La puberté précoce constitue un motif fréquent de consultation en endocrinologie pédiatrique. D´après une étude danoise menée par Teilmann, entre 1993 à 2001, à propos de 670 enfants présentant une puberté précoce, la prévalence a été estimée à 0,2% chez la fille et < 0,05 % chez le garçon [4]. Dans notre travail, la prévalence hospitalière était plus élevée (5,5 cas par an), ceci pourrait s´expliquer par l´inclusion des cas de puberté précoce dissociée. En Afrique, les études basées sur la population sont presque inexistantes. Cependant, dans une étude transversale menée par Abodo *et al*.[5], à propos de 8 cas de puberté précoce, il y avait une prédominance féminine, qui est rapportée également dans la littérature. Ceci a été également retrouvé dans notre série puisque 90% des cas était des filles.

Dans notre étude, les signes cliniques révélateurs les plus fréquents étaient le développement mammaire chez la fille et la pilosité pubienne avec augmentation de la verge chez le garçon. Ainsi, devant toute suspicion de puberté précoce, l´examen clinique doit rechercher les signes cliniques de la puberté avec estimation du stade pubertaire selon la classification de Tanner et Marshall [6]. La courbe de croissance doit être établie à la recherche d´une accélération staturale. Dans notre travail, nous avons noté une avance staturale moyenne de + 2,7 ± 0,8 DS. La présentation clinique, complétée par des examens complémentaires, permet de distinguer une puberté précoce pathologique d´une variante de la puberté normale. Les examens complémentaires confirment le diagnostic de puberté précoce en objectivant des sécrétions gonadiques de type pubertaire: chez le garçon, la testostérone est un marqueur fidèle de la maturation testiculaire, un taux > 0,3 ng / ml signe le début de la puberté [7]. Dans notre série, le taux moyen de testostérone était élevé à 3,38 ng/ml ± 0,9 ng/ml, ce qui est concordant aux données de la littérature. Chez la fille, le dosage de l´œstradiol par méthode radio-immunologique ne représente pas un outil fiable d´évaluation du début de la puberté. Ceci est dû à sa faible spécificité et ses fluctuations [7], d´où la nécessité de compléter systématiquement par une échographie pelvienne. Ainsi le démarrage de la puberté est marqué par une augmentation du volume ovarien > 1,5 ml [7] et surtout par un développement de l´utérus dont la longueur dépasse 3,7 cm avec une sensibilité à 88% et une spécificité à 95% [8]. L´existence d´un renflement fundique et d´une ligne de vacuité utérine témoigne d´une imprégnation œstrogénique significative [6]. Dans notre travail, chez les filles présentant une puberté précoce centrale, la longueur utérine moyenne a été estimée à 39 ± 4 mm ce qui rejoint les données de la littérature.

Sur le plan étiologique, la puberté précoce est le plus souvent d´origine centrale due à l´activation prématurée de l´axe hypothalamo-hypophyso-gonadique. Le taux de base de LH peut être utile pour le diagnostic de puberté précoce centrale, ainsi un taux > 0.6 UI/L (IFMA) ou > 0.3 UI/L (ICMA) signe le début de puberté [8, 9]. Dans notre série, le taux moyen de base de LH chez les filles présentant une puberté précoce centrale était augmenté à 2,57 ± 1,03 UI/L, ce qui est concordant avec les données de la littérature. Toutefois, le test de GnRH constitue le gold standard pour le diagnostic de puberté précoce centrale, en effet dans cette dernière, les taux de FSH et de LH augmentent avec un pic de LH qui devient supérieur au pic de FSH alors que dans la puberté précoce périphérique, les taux n´augmentent pas “réponse plate” [2]. Pour la majorité des études, un pic de LH > 5 UI /L indique une maturation de l´axe gonadotrope. Un pic de LH > 8UI /L constitue un critère puissant pour le diagnostic de puberté précoce centrale. Ceci a été retrouvé dans notre série puisque le taux moyen du pic de LH chez les filles présentant une puberté précoce centrale était de 17 UI/L. Ainsi dans les pubertés précoces centrales, les taux augmentent avec un rapport pic de LH/ pic de FSH > 1 (voir 0,66) [9], ceci a été également objectivé dans notre travail puisque le rapport moyen pic LH / pic FSH était de 1,30. En ce qui concerne le diagnostic étiologique, 38,60% des enfants de notre série avaient une puberté précoce pathologique. Cette dernière était d´origine centrale dans 41,02% des cas, en rapport avec une lésion du SNC chez la totalité des garçons (hamartome hypothalamique+) alors que chez la majorité des filles, la puberté précoce centrale était idiopathique.

Selon les principales données de la littérature, les fréquences respectives des formes organiques et idiopathiques de la puberté précoce centrale varient selon le sexe. La puberté précoce centrale idiopathique est rare chez le garçon (20-30%) et fréquente chez la fille (90%) [2]. Ceci a été également noté dans notre étude. Cette distinction permet de guider le choix des examens neuroradiologiques. Ainsi, l´imagerie par résonance magnétique (IRM) doit être pratiquée en première intention lorsqu´une cause organique est probable, notamment chez le garçon, chez les filles de moins de 6 ans avec un taux d´œstradiol plus ou moins élevé ou quand existent des signes neurologiques faisant craindre une pathologie du SNC [7]. En cas de puberté précoce isolée chez la fille de plus de 6 ans, le scanner cérébral est un bon outil de débrouillage suffisant pour l´exploration [7]. Les principales étiologies de la puberté précoce centrale sont résumées dans le Tableau 3 [10].

**Tableau 3:** les principales étiologies des pubertés précoces centrales [10]

Aucune anomalie dans le SNC	Avec lésion du SNC
Idiopathique causes génétiques: mutation activatrice KISS1 et KISS1R, mutation inactivatrice MKRN3 Exposition aux perturbateurs endocriniens secondaires, une exposition chronique aux stéroïdes sexuels: traitement tardif de l´HCS, à de syndrome de McCune- Albright Adoption internationale	Hamartome hypothalamique tumeurs: astrocytome, craniopharyngiome, épendymome, gliome optique ou hypothalamique,adénome hypophysaire sécrétant LH, pinéalome, dysgerminome malformations congénitales: kyste arachnoïdien, kyste suprasellaire, dysplasie septo-optique, hydrocéphalie, spina bifida, myéloméningocèle, malformation vasculaire, posthypophyse ectopique Maladies acquises: infections et processus inflammatoires du SNC: méningite, encéphalite sarcoïdose, tuberculose, irradiation cérébrale, asphyxie périnatale, traumatisme crânien

Notre travail présente une particularité, c´est la prédominance de la puberté précoce périphérique, ceci pourrait se justifier par le nombre élevé de cas du bloc enzymatique par 21 hydroxylase ou 11β hydroxylase mais également par des facteurs génétiques ou environnementaux qui restent à déterminer. Dans la puberté précoce périphérique, la sécrétion prématurée des stéroïdes sexuels peut être d´origine surrénalienne ou gonadique. La cause la plus fréquente étant hyperplasie congénitale des surrénales par bloc en 21 hydroxylase dans sa forme non classique [3]. Ceci a été également retrouvé dans notre étude. Les autres causes sont le syndrome de Mc Cune Albright, les tumeurs ovariennes ou testiculaires, la testotoxicose, les tumeurs surrénaliennes virilisantes ou féminisantes, et les causes iatrogènes [11]. L´hypothyroïdie périphérique ou syndrome de Van Wyk Grumbach reste une cause très rare de puberté précoce, cette dernière serait expliquée par le fait que l´augmentation de TSH aurait une action sur les récepteurs hormonaux de la FSH en raison de similitudes moléculaires entre les deux hormones [12]. Ces différentes étiologies suscitées ont été retrouvées également dans notre travail.

**Les limites:** la 1^ère^ limite est le fait que cette étude est rétrospective, cela implique l´existence de quelques données manquantes, la 2^e^ limite est la non réalisation de l´étude génétique devant des cas de puberté précoce idiopathique qui pourrait être en rapport avec des mutations génétiques. Cependant ces limites n´enlèvent rien à la qualité des renseignements fournis par cette étude.

## Conclusion

Notre étude confirme que la puberté précoce peut correspondre à une variante de la puberté normale ou à une puberté précoce pathologique. Dans cette dernière, la puberté précoce peut être d´origine centrale ou périphérique, surrénalienne ou gonadique. D´après les données de la littérature et les résultats de notre étude, devant toute puberté précoce centrale survenant chez le garçon, il va falloir demander systématiquement une imagerie cérébrale pour ne pas passer à côté une lésion du SNC. Et pour les enfants présentant une pseudo puberté précoce, il convient d´éliminer en 1^er^ lieu une HCS par bloc en 21 hydroxylase ou en 11 β hydroxylase.

**Figure 1:**
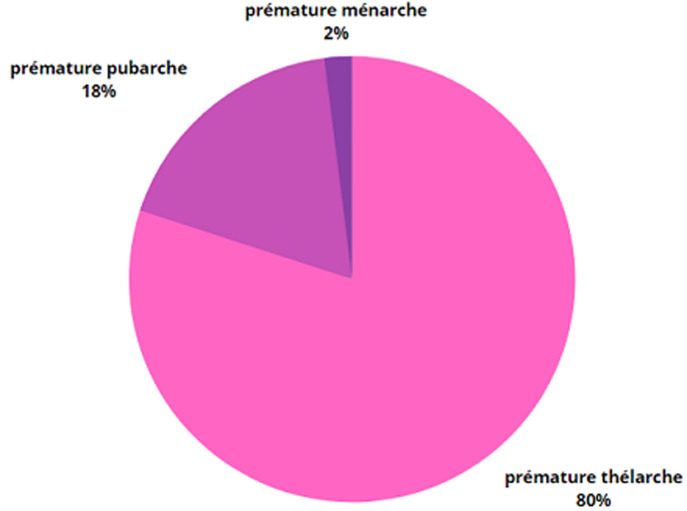
étiologies des pubertés dissociées

### Etat des connaissances sur le sujet

- La puberté précoce peut correspondre à une variante de la puberté normale ou à une puberté précoce pathologique;
- La puberté précoce pathologique peut être d´origine centrale ou périphérique surrénalienne ou gonadique;
- La puberté précoce a un impact sur la croissance et sur le psychique.

### Contribution de notre étude à la connaissance

- IRM cérébrale doit être systématique devant toute puberté précoce centrale (PPC) chez le garçon;
- Le test de GNRH constitue le gold standard pour poser le diagnostic de PPC;
- Devant toute pseudo-puberté précoce, il va falloir éliminer une HCS par bloc enzymatique en 21 hydroxylase.
